# Primary large cell prostate neuroendocrine carcinoma with central and nephrogenic diabetes insipidus

**DOI:** 10.1590/S1677-5538.IBJU.2019.0180

**Published:** 2020-07-31

**Authors:** Cem Basatac, Sezer Sağlam, Fatima Aktepe, Haluk Akpinar

**Affiliations:** 1 Istanbul Bilim University Department of Urology Istanbul Turkey Department of Urology, Istanbul Bilim University, Istanbul, Turkey; 2 Istanbul Bilim University Department of Medical Oncology Istanbul Turkey Department of Medical Oncology, Istanbul Bilim University, Istanbul, Turkey; 3 Group Florence Nightingale Hospitals Department of Pathology Istanbul Turkey Department of Pathology, Group Florence Nightingale Hospitals, Istanbul, Turkey

## INTRODUCTION

Neuroendocrine carcinoma of the prostate (PNEC) is rare and accounts for less than 0.5% of all prostate neoplasms (1). These tumors can be subdivided into small cell carcinomas, large cell neuroendocrine carcinomas (LCNEC) and carcinoid tumors based on the morphologic characteristics and proliferative index of tumors. PNEC is often diagnosed after long-lasting androgen deprivation therapy (ADT) for previous prostate adenocarcinoma (2). However, the pure form of this tumor is exceedingly rare and associated with aggressive behavior. Only a few cases with primary LCNEC have been published and its clinical course and treatment options remain unclear, yet (2, 3). In this study, we aim to report an unusual case of primary LCNEC with dexamethasone induced nephrogenic diabetes insipidus (NDI) during its management.

## CASE DESCRIPTION

A seventy-year-old male patient was admitted to our hospital with complaints of dysuria, hematuria, and stranguria lasting for four weeks. He also suffered from chronic constipation. He had a previous prostate biopsy in 2005 and pathology was benign prostate hyperplasia. There was no prior history of surgical procedures and significant comorbidities in his past medical history. Physical examination revealed abdominal distention but without significant tenderness. A large-sized fixed prostate that was completely adhering to the pelvic wall was detected on digital rectal examination. The serum creatinine and prostate-specific antigen values were 9.96mg/dL and 3.9ng/dL, respectively. Ultrasonography showed bilateral grade two hydronephrosis and suspicious mass invading through the bladder neck and trigone. A Foley catheter was inserted to achieve urinary drainage. However, oliguria was not resolved with conservative treatments and serum creatinine showed a gradually increasing trend.

**Figure 1:**
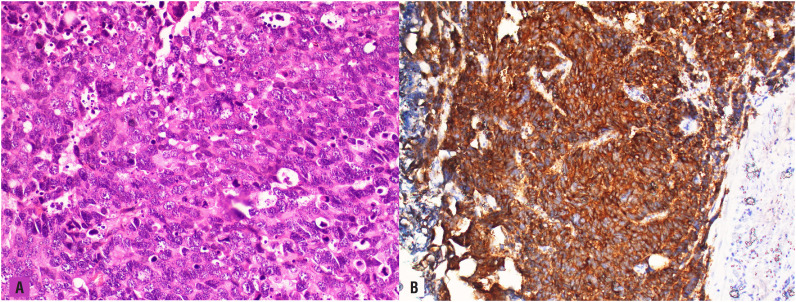
A) The tumor cells were large with a high nuclear/cytoplasmic ratio, severe atypia, and prominent nucleoli or salt and pepper appearance. Numerous mitotic figures and apoptotic bodies were observed (H&E, × 400). B) Immunohistochemically, the tumor cells were positive for synaptophysin (x 400).

**Figure 2:**
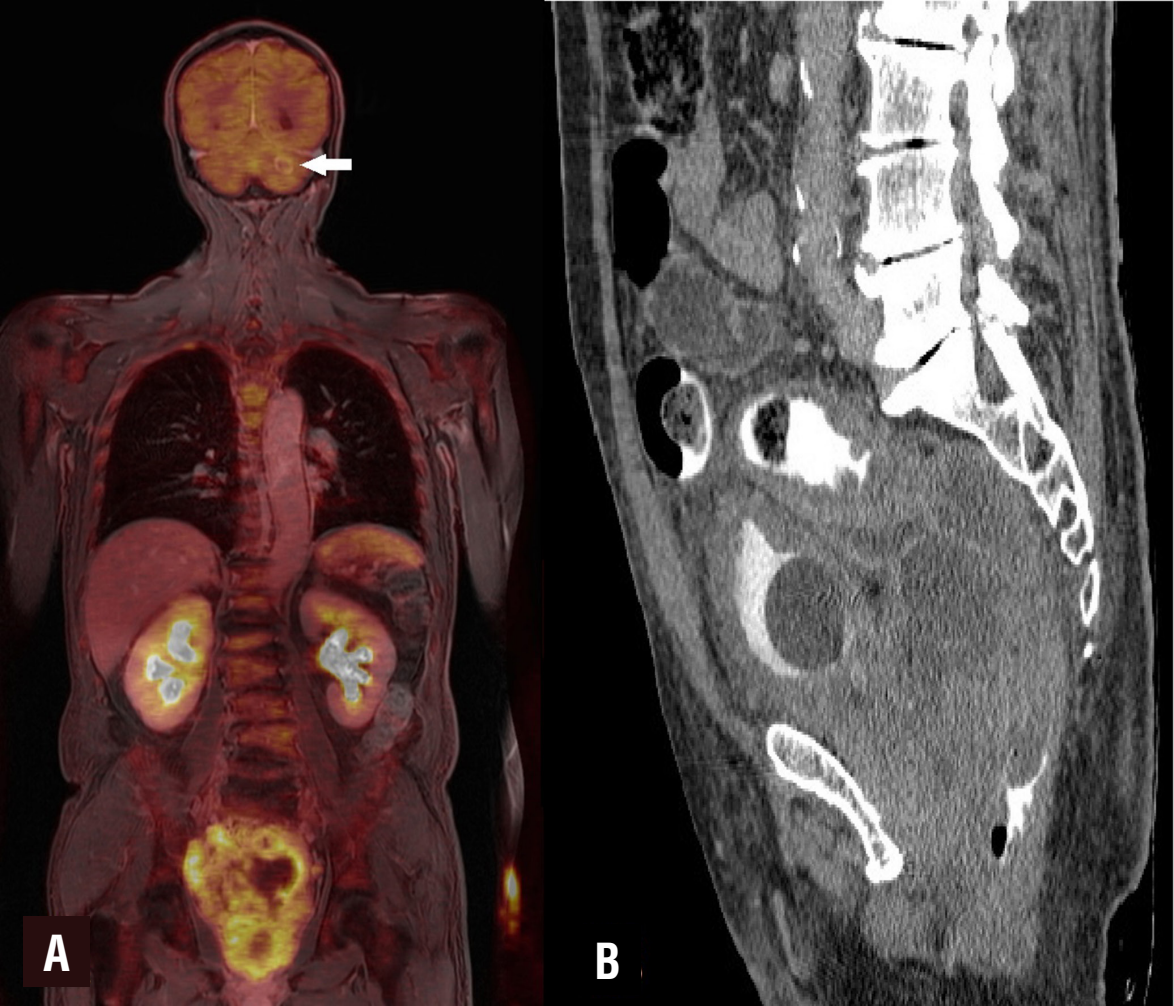
A) Coronal view of the tumor with PET-MR. (White arrow shows cerebellar metastases), B) Sagittal reconstruction of CT images showing tumor compression to the rectum.

A cystoscopy revealed massive prostatic mass invading bladder neck and trigone. A channel transurethral resection of the prostate was carried out in order to visualize obstructed ureteral orifices. After then, Resonance^®^ ureteral metal stents (Cook Urologic, Spencer, IN) were placed bilaterally under fluoroscopy guidance. A sextant transrectal prostate biopsy was also performed at the end of the procedure. Serum creatinine was normal at the postoperative 10^th^ day. Pathologic examination revealed LCNEC on both specimens. In tumor cell nuclei, some of them had prominent nucleoli and most of them had salt and pepper appearance (Figure-1). Geographic tumor necroses were also noted. Immunohistochemistry was used to ensure pathologic diagnoses. Whereas immunohistochemical stains showed diffusely positive staining for synaptophysin, tumor cell was not stained with the common prostate adenocarcinoma markers such as PSA, AMACR, ERG, and GATA3, CK7, CK20. Additionally, tumor cell proliferation as assessed by the Ki-67 index was so high (95%). PET-MR showed an irregular mass measuring 15x12x9 cm that originates from the prostate with higher SUVmax [10] and invading to the rectosigmoid colon causing subtotal obstruction. 18^F^- FDG uptake was positive in bilateral iliac and left paraaortic lymph nodes. A 6 cm metastatic lesion was noted on his posteromedial part of left femur diaphysis. In addition, PET-MR also showed brain metastases located at cerebellum and left parietal lobe (Figure-2).

We started classic systemic chemotherapy with cisplatin-etoposide. After the first session of the chemotherapy, his urine output increased significantly to 7000 cc/day with urine osmolality of 154 mOsm/kg suggesting central diabetes insipidus and it was normalized by desmopressin 120 mcg oral lyophilisate. Rectal stenosis due to mass effect of LCNEC was treated by a colorectal stent. Stereotactic body radiotherapy (SBRT) was performed to control the metastatic brain tumor growth using marginal doses of 20 Gy for metastatic lesions in the left parietal lobe and 24 Gy for the cerebellar lesion. However, 24 hours later there was an increased urine output again after a dexamethasone 8mg was started as a standard protocol at the same time of SBRT. It was unresponsive to treatment with desmopressin nasal spray, subcutaneous injection and regressed in 24 hours spontaneously when the dexamethasone was stopped after radiotherapy. Although primary tumor tended to shrink at the 3^rd^ months follow-up, a CT scan showed that primary, as well as the metastatic tumors, had progressed, and the patient died at the postoperative 7^th^ month due to multiple organ dysfunction.

## DISCUSSION

Large cell neuroendocrine carcinoma of the prostate can be considered as one of the most lethal and aggressive subsets of prostate cancers (4). Aggressive behavior of this tumor is usually related to loss of androgen receptor signaling pathways which causes castration-resistant prostate cancer. Therefore, differential diagnoses are essential with a precise pathologic examination from conventional prostate adenocarcinoma. LCNEC constitutes large arranged cell in nests and these tumor cells have also abundant cytoplasm and vesicular nucleoli with high mitotic activity by histologic examination. As in our case, geographic necrosis is often observed and it's accepted as an important histologic finding of this tumor type. In the immunohistochemical examination, PNEC usually does not express and stain with common prostate adenocarcinoma markers such as AR, P501S, PSMA, and PSA. But, it expresses and shows characteristically positive staining with neuroendocrine markers like chromogranin A, CD56, NSE, and synaptophysin. Among these, synaptophysin is considered as the most sensitive biomarker for PNEC (5). In addition, cell proliferation assessed by the Ki-67 index is significantly associated with poor prognoses in patients with prostate cancer (6). Whereas Ki-67 index is usually under 10% for prostate adenocarcinoma, this score is expected to be high usually greater than 50% for PNEC (5–7). In our case, the Ki-67 proliferation index of tumor cells were 95% and tumor cells showed strong and diffusely positive staining with synaptophysin that supported the neuroendocrine origin of the malignant cells.

Neuroendocrine carcinoma of the prostate is often diagnosed in patients who received long-lasting ADT for the treatment of previous prostate adenocarcinoma. It's shown that histologically focal neuroendocrine differentiation is found in prostate adenocarcinomas treated by ADT ranging from 10% to 80% (8). Apart from this relationship between neuroendocrine morphology and ADT, primary PNEC is as yet only limited to a few case reports in the current literature. Only a case series was reported by Evans et al. that evaluated clinicopathologic features of 7 cases with LCNEC of the prostate. Six of these tumors arose from prostate adenocarcinoma that was previously treated with hormone therapy. Only one case had de-novo LCNEC of the prostate. Platinum-based systemic chemotherapy was chosen in all but one patient. The one with clinically confirmed organ-confined disease underwent radical cystoprostatectomy after TUR-P showed LCNEC. However, all patients in this series with complete follow-up lost their lives due to widespread dissemination of their tumors with a mean survival of seven months (3).

Patients with LCNEC are more likely to present with severe lower urinary obstruction. Serum PSA value may be normal especially patients with primary LCNEC. Therefore, these tumors are most frequently diagnosed in the advanced stage. It is shown that most common metastatic sites are bone, brain, lung, and liver. A PET-MR might be a more suitable way to provide accurate clinical staging. Several studies showed that PET-MR had excellent diagnostic performance for the overall detection of the malignancies due to higher soft-tissue contrast of MRI with the highly sensitive evaluation of metabolism and molecular processes of PET (9). In addition, one of the most important questions is which is the most suitable way for the treatment of a malignant ureteral obstruction in these patients. We recommend metallic tumor stents (Resonance^®^) since several studies have shown that the metallic stents are more resistant to extrinsic compression than their plastic counterparts (10).

Treatment alternatives for LCNEC of the prostate included radical cystoprostatectomy, systemic chemotherapy, and radiation therapy. However, no certain consensuses have been made about the most appropriate option due to the lack of high volume studies, yet. Systemic chemotherapy should be chosen including platinum-based chemotherapy as it determined before in the treatment of neuroendocrine tumor of the lung (11). It is not clear in the literature whether radical cystoprostatectomy provides long term cancer-specific survival for organ-confined disease or not. There is too little data about whether radiation therapy is effective in LCNEC or not, and these findings usually exist from lung cancer series (11, 12). Therefore, such challenging cases should be discussed in multidisciplinary team meetings and care must be individualized depending on the tumor stage.

The other important scientific observation of our case report is the dexamethasone-induced NDI. We excluded other potential causes of NDI. To our knowledge, this is the second case report related to this topic after the first description by Toftegaard et al. (13). Dexamethasone was given as a standard protocol in patients treated by SBRT. Animal studies have shown that there was negative feedback between glucocorticoids and the ADH-secretion from the neurohypophysis (14). However, evidence-based, high-quality studies are needed to learn the exact role of glucocorticoids on the kidneys.

In conclusion, we strongly recommend that LCNEC should be kept in mind when patients have a prostate which is abnormally large in size and cause bilateral hydroureteronephrosis due to invasion through the bladder neck and trigone. In addition, the physician should not forget that widespread utilization of the potent androgen receptor signaling inhibitors can cause a rise in the incidence of the PNEC. Though clinical findings can be helpful for LCNEC, differential diagnosis currently depends on histopathologic evaluation. Therefore, tissue sampling using transrectal ultrasound guided prostate biopsy or TUR-P should not be neglected whenever clinical features are suggestive of LCNEC even in patients with a normal PSA value as in our case.
